# Reduced‐Dose Bendamustine as a First‐Line Treatment of Follicular Lymphoma Is Associated With Poorer Prognosis

**DOI:** 10.1002/cam4.71702

**Published:** 2026-03-08

**Authors:** Keisuke Tanaka, Atsushi Takahata, Yuma Noguchi, Keigo Okada, Tatsuya Saito, Junichi Mukae, Yuki Osada, Ken Suzuki, Daisuke Mizuchi, Koh Yamamoto, Takashi Kumagai, Gaku Oshikawa, Takehiko Mori, Shigeo Toyota, Masahide Yamamoto

**Affiliations:** ^1^ Department of Hematology Institute of Science Tokyo Hospital Tokyo Japan; ^2^ Department of Hematology Yokosuka Kyosai Hospital Kanagawa Japan; ^3^ Department of Hematology Japanese Red Cross Musashino Hospital Tokyo Japan; ^4^ Department of Hematology Ome Medical Center Tokyo Japan; ^5^ Department of Hematology Yokohama City Minato Red Cross Hospital Kanagawa Japan; ^6^ Department of Hematology Tokyo Teishin Hospital Tokyo Japan; ^7^ Department of Hematology The Fraternity Memorial Hospital Tokyo Japan

**Keywords:** bendamustine, dose‐reduction, follicular lymphoma

## Abstract

The dose of bendamustine used to treat previously untreated follicular lymphoma (FL) is sometimes reduced based on various clinical considerations, but the impact of such dose reductions on outcomes is unclear. We retrospectively analyzed 92 untreated FL patients treated with bendamustine at seven institutions in Japan. Dose reduction was defined as receiving less than 90% of the planned total dose of 1080 mg/m^2^ over six cycles. Patients with disease progression observed at ≤ 180 days of treatment initiation were defined as primary bendamustine‐refractory (PBR). The 3‐year overall survival (OS) and progression‐free survival (PFS) were 87.1% and 71.8%, respectively. Seven patients were classified as PBR, six of whom received bendamustine at full dose without dose reduction, and they had significantly worse OS with 3‐year OS of 38.1%. We excluded the PBR cases from subsequent analyses of the dose intensity's prognostic significance. The dose‐reduction group had a significantly lower CR rate (93.0% vs. 74.4%, *p* = 0.032). Similarly, the 3‐year PFS was significantly worse in the dose‐reduction group (86.8% vs. 68.1%, *p* = 0.005). In univariate and multivariate analyses, dose reduction was associated with inferior PFS. In an analysis limited to patients who completed all six courses, the CR rate was comparable between the two groups, but 3‐year PFS was significantly worse in the dose‐reduction group (86.8% vs. 68.7%, *p* = 0.041). Furthermore, even among patients who achieved CR, the 3‐year PFS tended to be poorer in the dose‐reduction group (85.7% vs. 72.7%, *p* = 0.062). These findings suggest that PBR cases are resistant to bendamustine itself regardless of the dose intensity. In contrast, among responders including those who achieved CR, dose reduction was associated with poorer PFS, indicating that maintaining treatment intensity is important for improving prognosis in the treatment of bendamustine‐responsive FL patients.

## 1 Introduction

Follicular lymphoma (FL) is the most common type of indolent B‐cell lymphoma, and it accounts for about one‐quarter of all malignant lymphomas [1]. The standard first‐line treatment for advanced‐stage FL is a combination of an anti‐CD20 monoclonal antibody and chemotherapy, such as CHOP (cyclophosphamide, doxorubicin, vincristine, and prednisone) or bendamustine, administered with either rituximab or obinutuzumab [2]. Among these regimens, bendamustine combined with rituximab or obinutuzumab has demonstrated promising efficacy and is increasingly used as a preferred first‐line therapy [3, 4].

Despite observations of bendamustine as a promising initial treatment of FL, this lymphoma remains incurable and recurrent relapses are frequent. The length of FL patients' progression‐free survival (PFS) tends to shorten with each successive relapse, and thus prolonging the duration of the response to the initial treatment is critical in order to improve the patients' overall survival (OS) [5, 6, 7]. Importantly, patients with FL who relapse within 24 months of their first‐line treatment exhibit significantly poorer OS, with earlier progression being associated with particularly adverse outcomes [8, 9, 10].

The treatment intensity, i.e., dose intensity, has been reported to play a significant role in the PFS of patients with previously untreated diffuse large B‐cell lymphoma (DLBCL) who received R‐CHOP therapy [11]. The impact of the intensity of R‐CHOP treatment in patients with FL was reported to be limited [12]. The effect of the treatment intensity of bendamustine has not been established. We conducted the present study to evaluate the impact of the treatment (dose) intensity of bendamustine on the prognosis of patients with previously untreated FL.

## 2 Material and Methods

### 2.1 Patients

We retrospectively analyzed the cases of the patients who had been diagnosed with FL histologically at one of seven hospitals in Japan and had been treated with a combination of an anti‐CD20 monoclonal antibody and bendamustine as their first‐line therapy during the period from May 2016 through February 2022. We excluded the cases of the patients with FL grade 3b and those who had not received anti‐CD20 monoclonal antibody. The study protocol was approved by the ethics committees of the Institute of Science Tokyo (approval no. M2018‐231). Informed consent was waived due to the retrospective nature of the study, which was approved by the Ethics Committee Institute of Science Tokyo.

### 2.2 Treatment and Response Assessment

The treatment with anti‐CD20 monoclonal antibody in combination with bendamustine was based on the regimen used in the GALLIUM trial [13]. The type of anti‐CD20 monoclonal antibody and the use of maintenance therapy were determined at the discretion of each patient's treating physician. Dose reductions of bendamustine, as well as treatment delays or discontinuation, were implemented at the discretion of the treating physician.

The patients' responses to the treatment had been evaluated with the use of PET/CT or CT (for cases in which PET/CT was not feasible); these evaluations were conducted at each institution without a central review. We defined the patients who exhibited disease progression at ≤ 180 days from the initiation of their bendamustine treatment as primary bendamustine‐refractory (PBR), and the patients who exhibited disease progression after the 180‐days timepoint were defined as showing relapse/progression.

### 2.3 Bendamustine Treatment Intensity

Bendamustine is generally administered at 90 mg/m^2^ for 2 consecutive days per course for a total of six courses, resulting in a planned total dose of 1080 mg/m^2^. We defined this study's ‘dose‐reduction’ group as the cases in which the patient's total dose was < 90% of the planned dose (972 mg/m^2^). We defined a ‘treatment interval extension’ group as the cases in which the treatment interval was ≥ 35 days; i.e., the interval between Day 1 of the first course and Day 1 of the sixth course exceeded 175 days.

### 2.4 Statistical Analysis

For the comparisons of patient groups, the χ^2^‐test or Fisher's exact test was used for categorical variables and the Mann–Whitney U‐test was used for continuous variables. The PFS and OS were estimated with the Kaplan–Meier method, and the group comparisons were made with a log‐rank test. The patients' OS was calculated from the date of treatment initiation to the date of death from any cause or the date of the patient's last visit. The PFS was calculated from the date of treatment initiation to the date of death from any cause, disease relapse or progression, or date of last visit. The univariate and multivariate analyses for PFS were conducted using the Cox proportional hazards model. The cumulative incidence of relapse (CIR) and non‐relapse mortality (NRM) were estimated from the date of treatment initiation, considering death and recurrence as competing events, respectively. All of the statistical analyses were conducted using EZR ver. 1.63, and probability (*p*)‐values < 0.05 were considered significant.

## 3 Results

### 3.1 Patient Characteristics

The characteristics of the patients with previously untreated FL are summarized in Table 1. A total of 92 cases were included in the analyses. The median age was 70 years, and 56.5% of the patients were female. Most of the cases were advanced‐stage with a high tumor burden according to the GELF criteria, and bone marrow infiltration was positive in about half of the cases. In 18 patients (19.6%), the initial dose of bendamustine was reduced due to advanced age (*n* = 11), comorbidities (*n* = 2), or other reasons (*n* = 5). Rituximab was the most commonly used anti‐CD20 monoclonal antibody (77.2%), and 13 patients (14.1%) received maintenance therapy (Table 1).

**TABLE 1:** The characteristics of the 92 patients with previously untreated follicular lymphoma.

Age, median (range)	70 (39–86)
Sex (male/female)	40/52
ECOG‐PS 0–1, *n* (%)	87 (94.6%)
Grade, *n* (%)	
1–2	66 (71.7%)
3A	22 (23.9%)
Unknown	4 (4.3%)
Clinical stage 3–4, *n* (%)	83 (90.2%)
PET/CT, *n* (%)	76 (82.6%)
Maximum SUVmax, median (range)	11.1 (2–29.8)
BM involvement, *n* (%)	48/87 (55.2%)
GELF criteria, *n* (%)	
≥ 3 sites each > 3 cm	32 (36.0%)
Mass > 7 cm	28 (31.5%)
Effusion	9 (10.1%)
B symptom	10 (11.2%)
Compression	27 (30.3%)
Cytopenia	8 (9.0%)
Leukemic phase	4 (4.5%)
Splenomegaly	5 (5.6%)
High tumor burden, *n* (%)	83 (90.2%)
Treatment	
Initial bendamustine dose reduction, *n* (%)	18 (19.6%)
Cause of dose reduction, *n*:	
Advanced age	11
Comorbidity	2
Other	5*
Anti‐CD20 antibody (O/R)	21/71
Maintenance treatment	13 (14.1%)

Abbreviations: BM, bone marrow; ECOG‐PS, Eastern Cooperative Oncology Group performance status; GELF, Groupe d’ Etude des Lymphomes Folliculaires; O/R, obinutuzumab/rituximab; PET, positron emission tomography; SUV, standardized uptake value.

* Impaired activities of daily living (*n* = 1), obesity (*n* = 2), unknown (*n* = 2).

### 3.2 Treatment Outcomes

**FIGURE 1:**
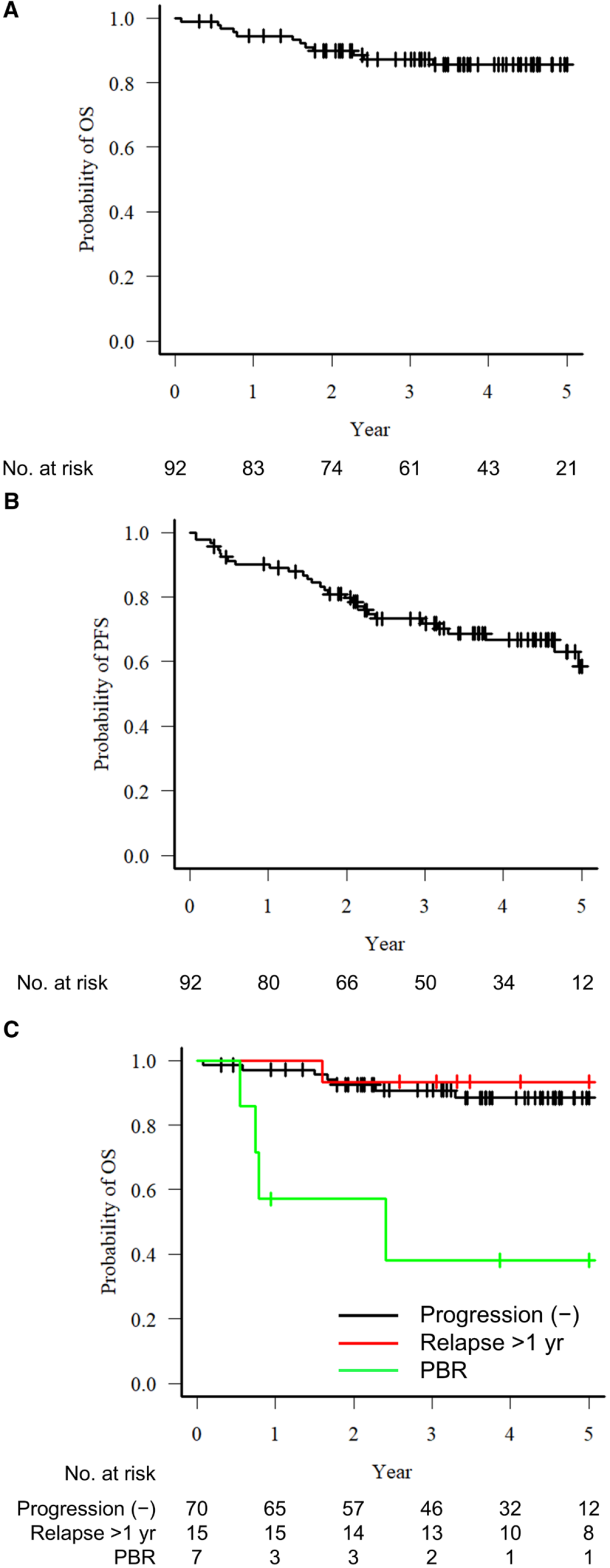
(A) The overall survival (OS) of the entire cohort of patients with previously untreated follicular lymphoma (FL) (*n* = 92). (B) The progression‐free survival (PFS) in the entire cohort. (C) The OS among the three patient groups: The patients without recurrence during the observation period (*black line*), those with recurrence occurring > 1 year after the initiation of treatment (*red line*), and those with recurrence that occurred at ≤ 180 days of treatment initiation (*green line*).

The median observation period was 3.7 years (range 0.1–7.6 years). Seventy patients (76.1%) achieved a complete response (CR) after the introduction of treatment. During the observational period, disease progression was observed in 22 patients, of whom seven experienced progression within 180 days; we classified them as PBR. Among the remaining 15 patients with relapse/progression, six experienced progressions within 24 months (POD24). In the total patient cohort, the 3‐year OS rate was 87.1% and the 3‐year PFS rate was 71.8% (Figure 1A,B). The patients in the PBR group had significantly worse OS compared to that of the other patients, with a 3‐year OS rate at 38.1% (Figure 1C). No cases of relapse were observed between 180 and 365 days after the initiation of treatment. Patients who experienced relapse more than 1 year after treatment initiation (relapse > 1 year) demonstrated overall survival comparable to that of patients who did not experience relapse during the observation period.

A total of 14 patients died during the study period. All of the deaths in the PBR group were due to disease progression. In contrast, among the non‐PBR cases, only one of the 10 deaths was attributable to disease progression. The remaining nine deaths were attributable to other causes (infection, *n* = 2; other malignancies, *n* = 1; unknown causes, *n* = 2; other causes, *n* = 4). The 3‐year CIR and 3‐year NRM were 14.6% and 7.6%, respectively.

### 3.3 PBR Patients

In the PBR group, one patient's bendamustine dose was reduced from the initiation of treatment, and none of the patients' bendamustine doses were reduced during the course of treatment prior to disease progression. As shown in Table S1, compared to the non‐PBR cases, the PBR patients exhibited significantly higher levels of lactate dehydrogenase (LDH) (non‐PBR 201 (132–772) U/L vs. PBR 330 (175–1707) U/L, *p* = 0.004) and maximum baseline standardized uptake values (SUVmax) (non‐PBR 10.2 (2.0–29.8) vs. PBR 19.5 (15.8–21.6), *p* = 0.008). The PBR group also had a significantly higher proportion of cases that presented with lesions measuring ≥ 7 cm (PBR 71.4% vs. non‐PBR 28.0%, *p* = 0.030). Re‐biopsies were performed in three cases at the time of progression, and histological transformation (HT) was confirmed in all three of these cases. The remaining four patients did not undergo biopsy at the first relapse.

### 3.4 Impact of Bendamustine Intensity

**FIGURE 2:**
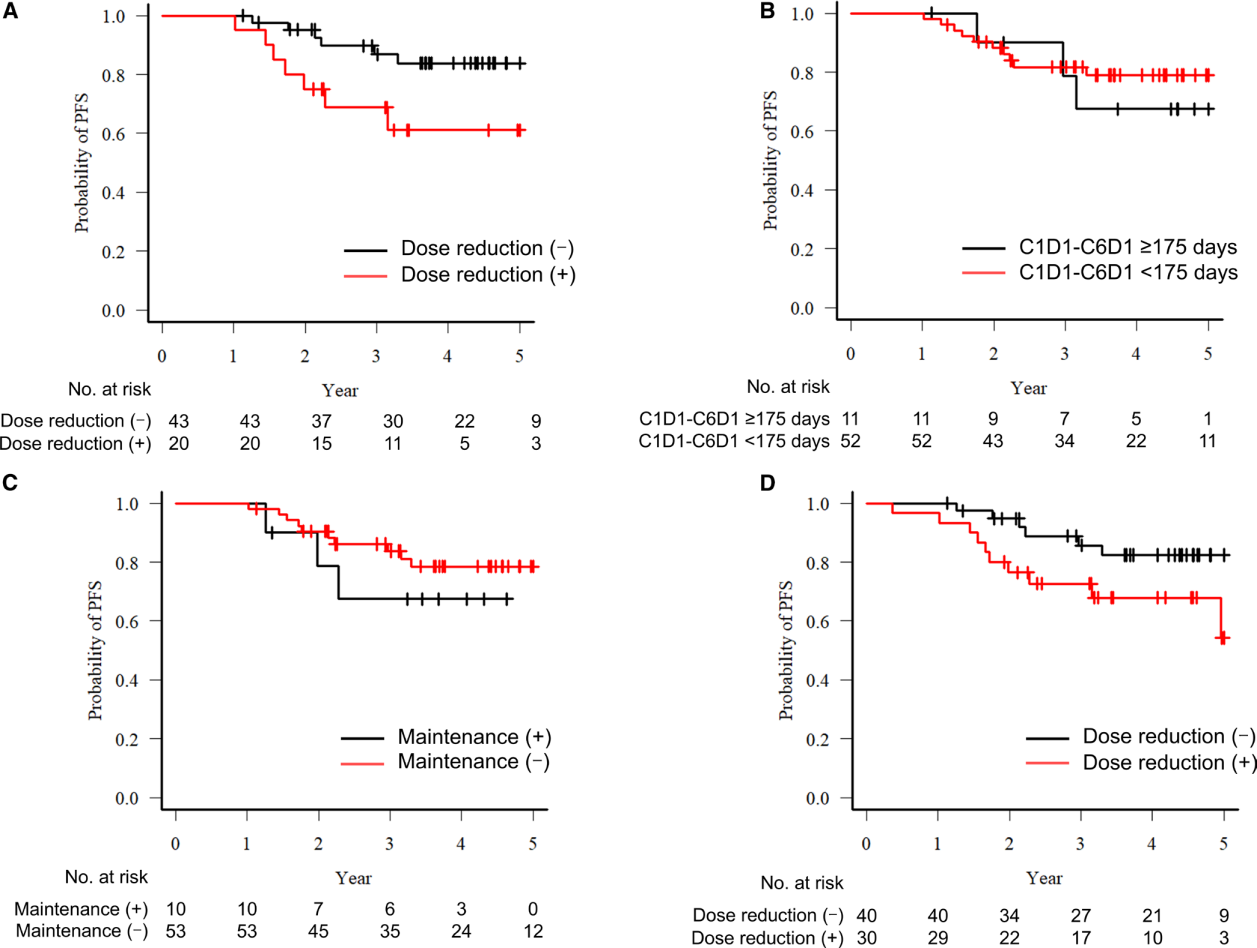
(A) The PFS stratified by the total dose of bendamustine among the patients who completed all six treatment cycles. *Black line:* The non‐dose‐reduction group. *Red line:* The dose‐reduction group. (B) The PFS stratified by the duration from Cycle 1 Day 1 (C1D1) to Cycle 6 Day 1 (C6D1) among the patients who completed all six treatment cycles. *Black line:* The patients with a treatment duration ≥ 175 days. *Red line:* The patients with a duration < 175 days. (C) The PFS according to maintenance therapy status in the patients who completed all six treatment cycles. *Black line:* The patients who received maintenance therapy. *Red line:* The patients who did not receive maintenance therapy. (D) The PFS stratified by the total dose of bendamustine among the patients who achieved a complete response (CR). *Black line:* The non‐dose‐reduction group. *Red line:* The dose‐reduction group.

We analyzed the impact of the dose intensity of the bendamustine treatment on the prognosis in 85 of the 92 patients (excluding the PBR patients). In 17 cases (20%), the bendamustine dose was reduced at the beginning of treatment, with the most common reason being advanced age. After starting the treatment, dose adjustment was applied in 11 cases and the bendamustine was discontinued in 22 cases, with cytopenia being the most frequent cause. Forty‐two patients were classified into the dose‐reduction group, which demonstrated significantly poorer 3‐year progression‐free survival compared with the non‐reduction group (86.8% vs. 68.1%, *p* = 0.005). A treatment response was observed in all analyzed cases, but the CR rate was significantly lower in the dose‐reduction group (93.0% vs. 74.4%, *p* = 0.032). Among the cases that completed all 6 courses of treatment, the dose‐reduction group showed a CR rate comparable to the non‐reduction group. However, PFS was significantly worse (*p* = 0.041, Figure 2A). This phenomenon was not observed when considering the presence or absence of treatment interval extension or maintenance therapy (Figure 2B,C). Even among the cases that achieved CR, the dose‐reduction group tended to have worse PFS (*p* = 0.062, Figure 2D). Univariate analysis identified male sex, poor PS, sIL‐2R > 3000 U/mL, and dose reduction as risk factors for poor PFS (Table 2). The type of anti‐CD20 antibody and the use of maintenance therapy did not have an impact on PFS. In the multivariate analysis, male sex, poor PS and dose reduction remained independent poor prognostic factors (Table 2).

**TABLE 2:** Univariate and multivariate analyses for progression‐free survival (PFS) in the patient series excluding the primary bendamustine‐refractory (PBR) patients.

	Univariate analysis		Multivariate analysis	
	HR (95% CI)	*p*	HR (95% CI)	*p*
Age > 60 years	1.430 (0.559–3.657)	0.456		
Male sex	2.358 (1.006–5.525)	0.048	2.487 (1.018–6.079)	0.046
PS 2–4	7.606 (2.208–26.20)	0.001	8.977 (2.412–33.41)	0.001
Grade 3a	0.815 (0.300–2.215)	0.688		
BM involvement	0.941 (0.384–2.308)	0.895		
Advanced clinical stage	0.784 (0.232–2.653)	0.696		
SUVmax > median	1.449 (0.557–3.768)	0.447		
Hb < 12 g/dL	0.785 (0.307–2.008)	0.613		
ALC < 1000/μL	1.003 (0.427–2.356)	0.995		
Alb ≤ 3.5 g/dL	0.902 (0.209–3.890)	0.89		
LDH > ULN	1.617 (0.690–3.791)	0.269		
sIL‐2R > 3000 U/mL	3.189 (1.353–7.516)	0.008	2.231 (0.904–5.507)	0.082
Total bendamustine dose < 972 mg/m^2^	3.585 (1.399–9.185)	0.008	3.588 (1.291–9.969)	0.014
RTX use	0.518 (0.198–1.354)	0.18		
Maintenance treatment	0.550 (0.202–1.497)	0.242		

Abbreviations: ALC, absolute lymphocyte count; BM, bone marrow; LDH, lactate dehydrogenase; PS, performance status; RTX, rituximab; sIL‐2R, soluble interleukin‐2 receptor; SUV, standardized uptake value; ULN, upper limit of normal.

**FIGURE 3:**
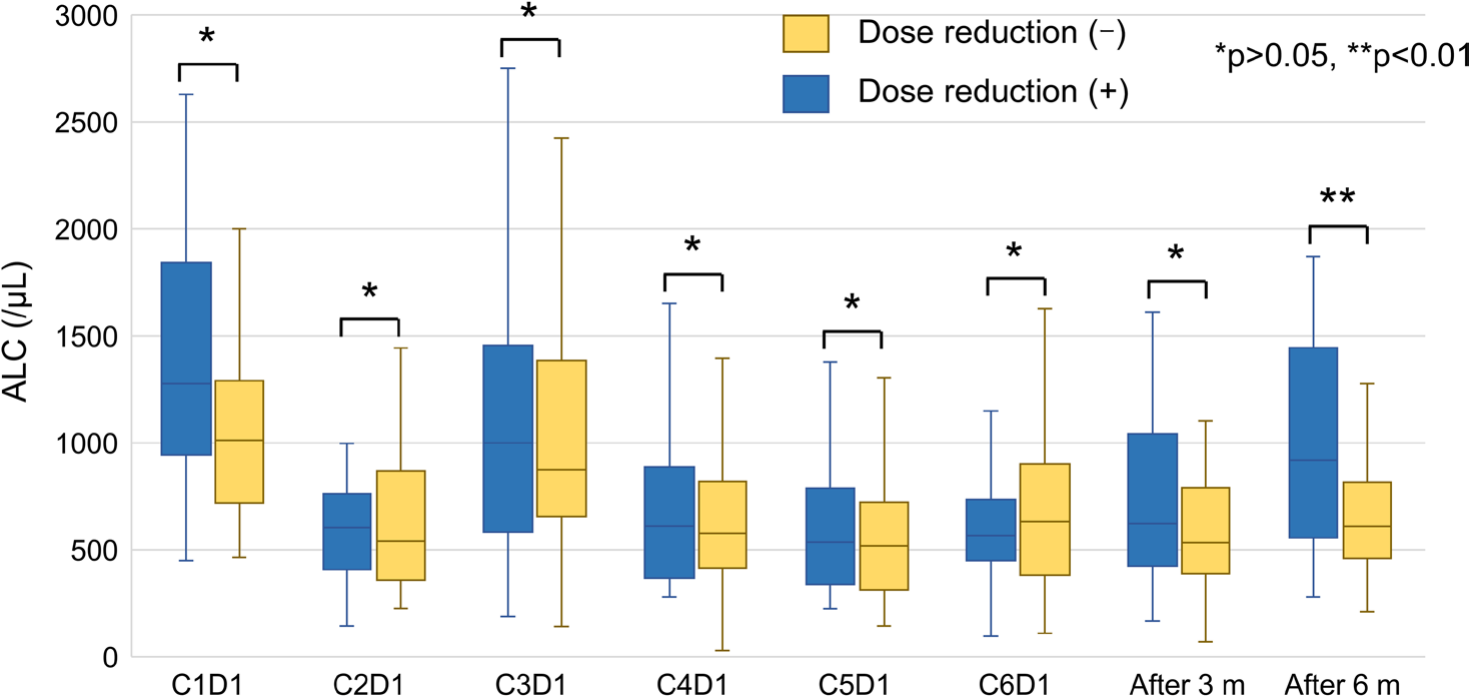
The transition of the absolute lymphocyte count (ALC) from the initiation of bendamustine treatment to 6 months after the completion of six treatment cycles. *Yellow bars:* The non‐dose‐reduction group. *Blue bars:* The dose‐reduction group.

Additionally, we analyzed the impact of bendamustine dose reduction on the absolute lymphocyte count (ALC). Among the patients who completed all six courses of treatment, there was no significant difference in ALC values between the dose‐reduction and non‐reduction groups from the initiation of therapy through the 3 months after the treatment completion. However, at 6 months post‐treatment, the ALC values were significantly higher in the dose‐reduction group (Figure 3), and the patients with ALC values > 800 at 6 months post‐treatment exhibited a significantly higher subsequent relapse rate compared to those with lower counts (30.3% vs. 10.6%, *p* = 0.041).

Regarding the transition of IgG levels, no significant difference in IgG level was observed between the groups up to 6 months after treatment completion, regardless of the application or non‐application of dose reduction (data not shown).

## 4 Discussion

The results of this study demonstrated that the efficacy of combination therapy with bendamustine and an anti‐CD20 monoclonal antibody was comparable to that of the rituximab‐bendamustine group in the GALLIUM study, indicating that this regimen is also a promising treatment option in real‐world clinical practice [4]. However, a small subset of the present patients experienced disease progression within 6 months after their treatment initiation, and these cases were associated with a poor prognosis. Since a reduction of the bendamustine dose was observed in only one of these patients, we considered these patients to be inherently refractory to bendamustine. Most of these patients had bulky disease or high baseline SUVmax values prior to treatment, and in all of the PBR patients who underwent a biopsy at their first relapse, HT was confirmed.

These results indicate that FL patients with such characteristics have a higher risk of early progression with HT when treated with bendamustine. Strati et al. reported that patients with such characteristics have a higher risk of HT and a poorer prognosis when treated with bendamustine [14]. Freeman et al. also reported that a substantial proportion of patients who experienced early disease progression after BR therapy underwent HT, and that elevated LDH levels were a risk factor for HT [15]. In our cohort, among PBR cases exhibiting these characteristics, some patients did not undergo biopsy of the lesion with the highest baseline SUVmax, suggesting the possibility that undiagnosed HT may have been present. According to Strati's report, even such cases do not necessarily lead to a poor prognosis when R‐CHOP is used as the initial therapy. Based on previous reports and our findings, FL with these characteristics should be treated with R‐CHOP as initial therapy, taking into consideration the risk of unrecognized DLBCL or early HT.

Our analyses excluding the PBR cases revealed that bendamustine dose reduction was one of the factors for poor PFS. The CR rate was significantly lower in the dose‐reduction group. In FL cases, the patient's post‐treatment response is a prognostic factor, and it has been suggested that the lower CR rate due to dose reduction contributes to the poor PFS [16, 17]. On the other hand, among our present study's patients who completed all six treatment courses, the PFS was poor in the dose‐reduction group although the dose‐reduction and non‐reduction groups' CR rates were comparable. In addition, even among the patients who achieved a CR, there was a tendency for poor prognosis in the patients whose bendamustine dose was reduced, suggesting that dose reduction also affects the long‐term prognosis after a CR is achieved.

There are only a few reports of investigations of the prognostic impact of bendamustine dose reduction [18, 19]. Bond et al. reported that the initial reduction of the bendamustine dose may be associated with worse prognosis, which is consistent with our present findings [19]. In contrast, Masamoto et al. reported no significant differences in the rates of CR or PFS between dose‐reduction and full‐dose groups [18]. However, in their report, the CR rate was lower in the dose‐reduction group (90.9% vs. 70.6%), and the relatively short observation period (~1 year) may have contributed to the lack of a significant between‐group difference in PFS.

Like the bendamustine dose, the treatment intervals affect the treatment intensity, but in the present FL cohort the prolongation of treatment intervals did not have an impact on prognosis. In light of this result, even in cases in which treatment is delayed due to adverse events, it is important to continue the patient's treatment without a dose reduction. Another factor that may influence prognosis is the implementation of maintenance therapy, but we observed that the use of maintenance therapy did not improve the prognosis in the patients who completed all six cycles of treatment. Another study showed no significant difference in treatment outcomes with or without maintenance therapy in patients who achieved a CR after rituximab and bendamustine treatment [20]. In the present study, the majority of patients who completed six treatment cycles achieved a CR; this may be why maintenance therapy did not have a significant impact on the patients' PFS. These findings suggest that maintaining the full cumulative dose of bendamustine, without dose reduction, is more important for improving the prognosis.

Bendamustine treatment is also known to induce marked lymphopenia during and/or after treatment, and we thus investigated the impact of the total cumulative dose of bendamustine on the ALC values of the patients who completed all six cycles of therapy. After bendamustine treatment, the ALC values decreased in both the dose‐reduction and non‐reduction groups, but after 6 months of treatment, the ALC values were significantly higher in the dose‐reduction group. This result was similar to a report suggesting that lymphopenia improves sooner with a decrease in the cumulative dose of bendamustine [21]. Our present analysis also revealed that lower ALC values at 6 months after treatment completion were associated with a higher recurrence rate. These findings indicate that the cumulative dose of bendamustine may influence not only the suppression of normal lymphocytes but also the control of lymphoma cells.

One of the limitations of this study is the unavailability of follicular lymphoma international prognostic index (FLIPI) and FLIPI2 data for the patients, which prevented the inclusion of these prognostic models in our analyses. These prognostic classifications were established based on patients who received non‐bendamustine‐based therapy, and the applicability of these classifications for patients treated with bendamustine‐containing regimens is unclear [22, 23]. In our analysis, factors that are part of the FLIPI prognostic classifications, i.e., Hb, age, LDH, clinical stage, and bone marrow involvement were not poor‐prognosis factors. Nagata et al. also reported that the FLIPI2 classification was not applicable for patients treated with bendamustine [24]. We thus suggest that the absence of these prognostic classification data is not important in our analysis of patients treated with bendamustine. More appropriate prognostic models are necessary for future examinations of bendamustine‐based regimens.

## 5 Conclusion

The overall survival of the present PBR (primary bendamustine‐refractory) patients with follicular lymphoma was extremely poor, indicating the necessity of careful selection of the initial treatment for such patients. Our non‐PBR patients demonstrated favorable prognoses, but since the total dose of bendamustine impacts patient outcomes, it is important to administer bendamustine without dose reduction.

## Author Contributions

K.T., M.Y., designed the research, performed statistical analysis, and wrote the manuscript. All authors collected clinical data and reviewed the manuscript critically. All authors approved the final manuscript.

## Funding

The authors received no financial support for the research, authorship, and/or publication of this article.

## Ethics Statement

The study protocol was approved by the ethics committees of the Institute of Science Tokyo (approval no. M2018‐231).

## Consent

Informed consent was waived due to the retrospective nature of the study, which was approved by the Ethics Committee Institute of Science Tokyo.

## Conflicts of Interest

T.S., received honoraria from Chugai. Y.O., received honoraria from Chugai. K.Y., received honoraria from Chugai. G.O., received honoraria from Chugai. T.M., received honoraria from Chugai, Kyowa kirin, Symbio and grant from Chugai, Kyowa kirin. M.Y., received honoraria from Chugai, Kyowa kirin, Symbio. The remaining authors have no relevant conflicts of interest to declare.

## Supporting information


**Table S1:** The characteristics of the non‐PBR and PBR patients.

## Data Availability

The data that support the findings of this study are available from the corresponding author upon reasonable request.

## References

[1] Cancer Statistics , “Cancer Information Service, National Cancer Center, Japan ‐ National Cancer Registry, Ministry of Health, Labour and Welfare,”.

[2] National Comprehensive Cancer Network , “NCCN Clinical Practice Guidelines in Oncology: B‐Cell Lymphomas. Version 2.2025,” https://www.nccn.org.

[3] M. J. Rummel , N. Niederle , G. Maschmeyer , et al., “Bendamustine Plus Rituximab Versus CHOP Plus Rituximab as First‐Line Treatment for Patients With Indolent and Mantle‐Cell Lymphomas: An Open‐Label, Multicentre, Randomised, Phase 3 Non‐Inferiority Trial,” Lancet 381, no. 9873 (2013): 1203–1210, 10.1016/S0140-6736(12)61763-2. 23433739

[4] W. Hiddemann , A. M. Barbui , M. A. Canales , et al., “Immunochemotherapy With Obinutuzumab or Rituximab for Previously Untreated Follicular Lymphoma in the GALLIUM Study: Influence of Chemotherapy on Efficacy and Safety,” Journal of Clinical Oncology 36, no. 23 (2018): 2395–2404, 10.1200/JCO.2017.76.8960. 29856692

[5] J. Liu , Y. Hu , L. Zhao , et al., “Management and Clinical Outcomes of Follicular Lymphoma Across Continuous Lines of Treatments: A Retrospective Analysis in China,” Frontiers in Oncology 13 (2023): 1264723, 10.3389/fonc.2023.1264723. 37941553 PMC10628462

[6] C. Batlevi , F. Sha , A. Alperovich , et al., “Follicular Lymphoma in the Modern Era: Survival, Treatment Outcomes, and Identification of High‐Risk Subgroups,” Blood Cancer Journal 10, no. 7 (2020): 74, 10.1038/s41408-020-00340-z. 32678074 PMC7366724

[7] A. R. Delgado , L. Magnano , M. M. Velazquez , et al., “Response Duration and Survival Shorten After Each Relapse in Patients With Follicular Lymphoma Treated in the Rituximab Era,” British Journal of Haematology 184, no. 5 (2019): 753–759, 10.1111/bjh.15708. 30515755

[8] C. Casulo , M. Byrtek , K. L. Dawson , et al., “Early Relapse of Follicular Lymphoma After Rituximab Plus Cyclophosphamide, Doxorubicin, Vincristine, and Prednisone Defines Patients at High Risk for Death: An Analysis From the National LymphoCare Study,” Journal of Clinical Oncology 33, no. 23 (2015): 2516–2522, 10.1200/JCO.2014.59.7534. 26124482 PMC4879714

[9] C. E. Weibull , T. Wasterlid , B. E. Wahlin , et al., “Survival by First‐Line Treatment Type and Timing of Progression Among Follicular Lymphoma Patients: A National Population‐Based Study in Sweden,” Hema 7, no. 3 (2023): e838, 10.1097/HS9.0000000000000838. PMC9953041 36844185

[10] J. F. Seymour , R. Marcus , A. Davies , et al., “Association of Early Disease Progression and Very Poor Survival in the GALLIUM Study in Follicular Lymphoma: Benefit of Obinutuzumab in Reducing the Rate of Early Progression,” Haematologica 104, no. 6 (2019): 1202–1208, 10.3324/haematol.2018.209015. 30573503 PMC6545851

[11] E. J. Bataillard , C. Y. Cheah , M. J. Maurer , A. Khurana , T. A. Eyre , and T. C. El‐Galal , “Impact of R‐CHOP Dose Intensity on Survival Outcomes in Diffuse Large B‐Cell Lymphoma: A Systematic Review,” Blood Advances 5, no. 9 (2021): 2426–2437, 10.1182/bloodadvances.2021004665. 33961018 PMC8114545

[12] K. Wudhikarn , B. J. Smith , A. M. Button , et al., “Relationships Between Chemotherapy, Chemotherapy Dose Intensity and Outcomes of Follicular Lymphoma in the Immunochemotherapy Era: A Report From the University of Iowa/Mayo Clinic SPORE Molecular Epidemiology Resource,” Leukemia & Lymphoma 56, no. 8 (2015): 2365–2372, 10.3109/10428194.2014.994206. 25530345 PMC4530103

[13] R. Marcus , A. Davies , K. Ando , et al., “Obinutuzumab for the First‐Line Treatment of Follicular Lymphoma,” New England Journal of Medicine 377 (2017): 1331–1344, 10.1056/NEJMoa1614598. 28976863

[14] P. Strati , M. A. Ahmed , N. H. Fowler , et al., “Pre‐Treatment Maximum Standardized Uptake Value Predicts Outcome After Frontline Therapy in Patients With Advanced Stage Follicular Lymphoma,” Haematologica 105, no. 7 (2020): 1907–1913, 10.3324/haematol.2019.230649. 31601688 PMC7327641

[15] C. L. Freeman , R. Kridel , A. A. Moccia , et al., “Early Progression After Bendamustine‐Rituximab Is Associated With High Risk of Transformation in Advanced Stage Follicular Lymphoma,” Blood 134, no. 9 (2019): 761–764. 31300404 10.1182/blood.2019000258

[16] J. Trotman , S. F. Barrington , D. Belada , et al., “Prognostic Value of End‐Of‐Induction PET Response After First‐Line Immunochemotherapy for Follicular Lymphoma ‐ GALLIUM: Secondary Analysis of a Randomised, Phase 3 Trial,” Lancet Oncology 19, no. 11 (2018): 1530–1542, 10.1016/S1470-2045(18)30618-1. 30309758

[17] J. Trotman , S. Luminari , S. Boussetta , et al., “Prognostic Value of PET‐CT After First‐Line Therapy in Patients With Follicular Lymphoma: A Pooled Analysis of Central Scan Review in Three Multicentre Studies,” Lancet Haematol 1, no. 1 (2014): e17–e27, 10.1016/S2352-3026(14)70008-0. 27030064

[18] Y. Masamoto , A. Shimura , and M. Kurokawa , “Reduced Bendamustine for Elderly Patients With Follicular Lymphoma,” Annals of Hematology 101, no. 3 (2022): 713–715, 10.1007/s00277-021-04576-y. 34152427 PMC8216086

[19] D. A. Bond , Y. Huang , A. S. Ruppert , et al., “Retrospective Analysis of Bendamustine and Rituximab Use in Indolent and Mantle Cell Non‐Hodgkin Lymphoma Based on Initial Starting Dose,” Leukemia & Lymphoma 58, no. 7 (2017): 1589–1597, 10.1080/10428194.2016.1253835. 27838951 PMC5499523

[20] B. T. Hill , L. Nastoupil , A. M. Winter , et al., “Maintenance Rituximab or Observation After Frontline Treatment With Bendamustine‐Rituximab for Follicular Lymphoma,” British Journal of Haematology 184, no. 4 (2019): 524–535, 10.1111/bjh.15720. 30575016 PMC6486816

[21] K. Manos , L. Churilov , A. Grigg , et al., “Infection Risk and Antimicrobial Prophylaxis in Bendamustine‐Treated Patients With Indolent Non‐Hodgkin Lymphoma: An Australasian Lymphoma Alliance Study,” British Journal of Haematology 205, no. 1 (2024): 146–157, 10.1111/bjh.19407. 38485116

[22] P. Solal‐Céligny , P. Roy , P. Colombat , et al., “Follicular Lymphoma International Prognostic Index,” Blood 104, no. 5 (2004): 1258–1265, 10.1182/blood-2003-12-4434. 15126323

[23] M. Federico , M. Bellei , L. Marcheselli , et al., “Follicular Lymphoma International Prognostic Index 2: A New Prognostic Index for Follicular Lymphoma Developed by the International Follicular Lymphoma Prognostic Factor Project,” Journal of Clinical Oncology 27, no. 27 (2009): 4555–4562, 10.1200/JCO.2008.21.3991. 19652063

[24] H. Nagata , T. Tsukamoto , T. Kobayashi , et al., “The Real‐World Efficacy and Safety of Frontline Therapy of Obinutuzumab Plus Bendamustine for Untreated High‐Tumor‐Burden Follicular Lymphoma,” International Journal of Clinical Oncology 30, no. 3 (2025): 593–603, 10.1007/s10147-025-02691-8. 39776016

