# Supplementary material for: Reduced‐Dose Bendamustine as a First‐Line Treatment of Follicular Lymphoma Is Associated With Poorer Prognosis
Source: Cancer Med. 2026 Mar 8;15(3):e71702. doi: 10.1002/cam4.71702 (PMC12967496; doi:10.1002/cam4.71702)
Supplement: Supplementary file 1 — Table S1: The characteristics of the non‐PBR and PBR patients. [file CAM4-15-e71702-s001.docx]

Table S1. The characteristics of the non-PBR and PBR patients

|  | Non-PBR (n=85) | PBR (n=7) | p-value |
| --- | --- | --- | --- |
| Age, median (range) | 69 (39-86) | 72 (59-81) | 0.338 |
| Sex (male/female) | 36/49 | 4/3 | 0.463 |
| ECOG-PS 0-1, n (%) | 81 (95.3%) | 6 (85.7%) | 0.333 |
| GELF criteria, n(%) |  |  |  |
| ≥3 sites each >3cm | 29 (35.4%) | 3 (42.9%) | 0.699 |
| Mass>7cm | 23 (28.0%) | 5 (71.4%) | 0.03 |
| Effusion | 9 (11.0%) | 0 (0%) | 1 |
| B symptom | 10 (12.2%) | 0 (0%) | 1 |
| Compression | 24 (29.3%) | 3 (42.9%) | 0.429 |
| Cytopenia | 8 (9.8%) | 0 (0%) | 1 |
| Leukemic phase | 4 (4.9%) | 0 (0%) | 1 |
| Splenomegaly | 4 (4.9%) | 1 (14.3%) | 0.343 |
| High tumor burden, n(%) | 76 (89.4%) | 7 (100%) | 1 |
| LDH (U/L) | 201 (132-772) | 330 (175-1707) | 0.004 |

PBR: primary bendamustine-refractory, ECOG-PS: Eastern Cooperative Oncology Group performance status, GELF: Groupe d' Etude des Lymphomes Folliculaires, LDH: lactate dehydrogenase
